# Semi-Scavenging Poultry as Carriers of Avian Influenza Genes

**DOI:** 10.3390/life12020320

**Published:** 2022-02-21

**Authors:** A T M Badruzzaman, Md. Masudur Rahman, Mahmudul Hasan, Mohammed Kawser Hossain, Asmaul Husna, Ferdaus Mohd Altaf Hossain, Mohammed Giasuddin, Md Jamal Uddin, Mohammad Rafiqul Islam, Jahangir Alam, Seong-Kug Eo, Folorunso Oludayo Fasina, Hossam M. Ashour

**Affiliations:** 1 Faculty of Veterinary, Animal and Biomedical Sciences, Sylhet Agricultural University, Sylhet 3100, Bangladesh; badruzzaman.sau@gmail.com (A.T.M.B.); rahmanmm.dpp@sau.ac.bd (M.M.R.); kawser.dst@sau.ac.bd (M.K.H.); asmaulhusna.dpp@sau.ac.bd (A.H.); ferdaus.dps@sau.ac.bd (F.M.A.H.); 2 National Reference Laboratory for Avian Influenza, Bangladesh Livestock Research Institute, Savar, Dhaka 1340, Bangladesh; mh.dvmru@yahoo.com (M.H.); mgias04@yahoo.com (M.G.); 3 ABEx Bio-Research Center, East Azampur, Dhaka 1230, Bangladesh; hasan800920@gmail.com; 4 Graduate School of Pharmaceutical Sciences, College of Pharmacy, Ewha Womans University, Seoul 03760, Korea; 5 Livestock Division, Bangladesh Agricultural Research Council, Farmgate, Dhaka 1215, Bangladesh; mrislam210@hotmail.com; 6 Animal Biotechnology Division, National Institute of Biotechnology, Savar, Dhaka 1349, Bangladesh; alamjahan2003@yahoo.com; 7 College of Veterinary Medicine and Bio-Safety Research Institute, Chonbuk National University, Iksan 54596, Korea; vetvirus@jbnu.ac.kr; 8 Emergency Centre for Transboundary Animal Diseases, Food and Agriculture Organization of the United Nations (ECTAD-FAO), United Nations Office in Nairobi (UNON), UN Avenue, Gigiri, Nairobi 00100, Kenya; folorunso.fasina@fao.org; 9 Department of Veterinary Tropical Diseases, University of Pretoria, Onderstepoort 0110, South Africa; 10 Department of Integrative Biology, College of Arts and Sciences, University of South Florida, St. Petersburg, FL 33701, USA

**Keywords:** avian influenza, molecular detection, semi-scavenging ducks

## Abstract

Ducks are the natural reservoir of influenza A virus and the central host for the avian influenza virus (AIV) subtype H5N1, which is highly pathogenic. Semi-scavenging domestic ducks allow for the reemergence of new influenza subtypes which could be transmitted to humans. We collected 844 cloacal swabs from semi-scavenging ducks inhabiting seven migratory bird sanctuaries of Bangladesh for the molecular detection of avian influenza genes. We detected the matrix gene (M gene) using real-time RT–PCR (RT–qPCR). Subtyping of the AIV-positive samples was performed by RT–qPCR specific for H5, H7, and H9 genes. Out of 844 samples, 21 (2.488%) were positive for AIV. Subtyping of AIV positive samples (*n* = 21) revealed that nine samples (42.85%) were positive for the H9 subtype, five (23.80%) were positive for H5, and seven (33.33%) were negative for the three genes (H5, H7, and H9). We detected the same genes after propagating the virus in embryonated chicken eggs from positive samples. Semi-scavenging ducks could act as carriers of pathogenic AIV, including the less pathogenic H9 subtype. This can enhance the pathogenicity of the virus in ducks by reassortment. The large dataset presented in our study from seven areas should trigger further studies on AIV prevalence and ecology.

## 1. Introduction

Influenza viruses pose a persistent public health threat for the global population. These viruses cause mild to severe illness with various respiratory and gastrointestinal symptoms. The World Health Organization (WHO) estimates that around one billion people are infected with influenza viruses every year [1]. Currently, Type A avian influenza viruses (AIVs) and Type B AIVs are the most prevalent in the human population. Type A AIVs, particularly those with a highly pathogenic nature, are responsible for medium to large-scale epizootics. Zoonotic influenza can spread rapidly causing dreadful economic, food security, and human health consequences [2,3,4]. Highly pathogenic avian influenza (HPAI) viruses of the H5N1 subtype have led to significant mortality in humans [5]. In 1996, the H5N1 subtype emerged in Guangdong, China [6], and was subsequently spread to other countries in the world. As of April 2021, the records of the WHO indicated 862 human H5N1 cases from 17 different countries, including Bangladesh [7,8]. Other influenza subtypes such as H5N2, H5N3, H5N5, H5N6, H5N8, H5N9, H7N1, H7N2, H7N3, H7N7, H7N8, H7N9, and H9N2 have been isolated from different parts of the world [9,10]. It was hypothesized that the highly pathogenic H5 and H7 viruses emerged in infected chicken as a result of genetic mutations (antigenic drift or antigenic shift) in low pathogenic avian influenza (LPAI) viruses [11,12]. AIVs replicate in the respiratory and intestinal epithelial cells of their natural reservoirs (wild waterfowl, gulls, dabbling ducks, geese, seabirds, and shorebirds) [13,14,15,16]. They are excreted through feces into the environment [17,18]. Migratory wild birds may have contributed to the spread of AIVs along the Central Asian Flyway and on a global level [19,20].

Many factors are responsible for the establishment, persistence, and spread of HPAI viruses, including crowded animal markets, poor farming conditions, trade practices of the poultry industry, transportation of poultry over long distances, and poor biosecurity practices [21,22,23,24,25,26,27,28,29]. Currently, several East Asian countries (Bangladesh, China, Indonesia, and Viet Nam), as well as Egypt, are considered to be endemic for HPAI viruses [30,31]. The problem is exacerbated when various subtypes of HPAI and LPAI viruses cocirculate in different species of birds [32,33,34,35,36,37,38]. Domestic ducks appear to serve as intermediary mixing vessels for several subtypes of influenza viruses following acquisition from migratory water birds [39,40,41]. Thereafter, viruses are transmitted from carrier ducks to chickens leading to potential outbreaks and public health events [42,43].

Bangladesh has tens of millions of ducks and ranks third in duck populations worldwide (out of a total global poultry population of more than 300 million) [44]. During winter, ducks are allowed to scavenge in migratory bird sanctuaries, increasing the risk of AIV infections from overwintering waterfowls and the subsequent transmission to domestic chickens [45]. Thus, the complex epidemiology of avian influenza in Bangladesh involves reassortment and exchanges of genetic materials among viruses with the resultant reemergence of new subtypes [46,47]. There are many migratory bird sanctuaries and large duck populations in the Sylhet Division of Bangladesh. However, surveillance data of AIVs in these preserved areas are scarce. Therefore, in this study, we collected biological samples (cloacal swabs) from semi-scavenging domestic ducks to determine the prevalence of the circulating subtypes of AIVs in seven contiguous wintering sites in Sylhet, Bangladesh. The goal is to obtain data that can guide policy decisions on animal health and zoonosis to minimize biosecurity risks and control any future outbreaks of AIVs.

## 2. Materials and Methods

### 2.1. Location

**Figure 1:**
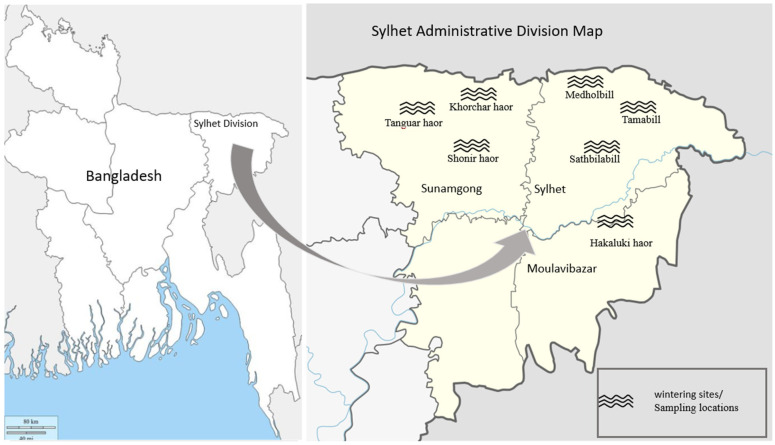
Map of Sylhet (highlighting sampling locations from the seven wintering sites).

Random surveillance for AIV in ducks was conducted in seven geographically diverse sites in Sylhet division, Bangladesh. The seven sampling sites fell within three different administrative areas including (1) Sylhet(24°36′ to 25°11′ N and 91°38′ to 92°30′ E); (2) Sunamgong (24°34′ to 25°12′ N and 90°56′ to 91°49′ E); (3) Moulavibazar (24°08′ to 24°29′ N and 91°36′ to 92°17′ E) (Figure 1). Sylhet division is bordered on the north by the Meghalaya, Assam, and Tripura states (India); on the east and south by three of the Bangladesh divisions; on the southwest by Chittagong; and on the west by Dhaka and Mymensingh. Seven wintering sites (Figure 1) with natural haors and bills were selected: Tamabill, Sathbilabill, Medholbill, Tanguar haor, Khorchar haor, Shonir haor, and Hakaluki haor. A bill/haor is a wetland ecosystem in the northeastern part of Bangladesh (bowl- or saucer-shaped shallow depression). Sampling was targeted at these sites because of the absence of recent records of AIV outbreaks. During the winter season (December to February) of every year, migratory waterfowls visit the selected haors, bills, and their water bodies. Domestic semi-scavenging ducks in the vicinities share these water bodies with the migratory birds during this period.

### 2.2. Field Sampling Procedures

Cloacal swabs were collected from randomly selected apparently healthy domestic semi-scavenging ducks that are older than one year (*n* = 844) at the seven sites for three successive seasons (summer: *n* = 280; rainy season: *n* = 274; and winter: *n* = 290; Table 1). Permissions for collecting duck were obtained from farm owners and households. After collection, sterile samples were added to 1 mL of PBS in sterile 1.5 mL Eppendorf tubes. All samples were preserved at 4 °C and dispatched (within 48 h) to the National Reference Laboratory for Avian Influenza (NRLAI), Bangladesh. Samples were stored at −80 °C until tested. All experimental procedures related to animal handling and welfare were approved by the Animal Care and Ethics Committees of Sylhet Agricultural University, Bangladesh.

### 2.3. Viral RNA Extraction, Molecular Detection, and Subtyping of AIV Isolates

Samples were screened for the presence of AIV by targeting the viral M gene. Cloacal swab samples were pooled in fives (200 µL from each sample to make 1 mL from 5 pooled samples), leading to a total of 169 pooled samples. Total RNA was harvested from the 169 pooled samples using the QIAGEN RNeasy Mini spin column (Hilden, Germany), in accordance with the manufacturer’s instructions. AIV-positive samples were identified by detecting the M gene using RT–qPCR in which the AgPath-ID™ One-Step RT–PCR kit (Thermo Fisher Scientific, Waltham, MA, USA) was used. M-gene-positive samples were subjected to total RNA extraction. A one-step RT–qPCR assay was used to detect type A AIV-positive samples, which were subtyped for the H5, H7, and H9 genes using specific primers and probes [48,49,50]. Positive controls for H5, H7, and H9 were supplied by the Australian Centre for Disease Preparedness (ACDP), CSIRO, Australia. The primer and probe sequences used in the study are shown in Table 2.

### 2.4. Virus Isolation in Embryonated Chicken Eggs

For each RT–qPCR-positive sample, virus isolation was attempted to determine the presence of live virus using hemagglutination activity (HA) [51]. Processed and filtered suspensions of cloacal swabs from RT–qPCR-positive samples were inoculated into 10-day-old specific-pathogen-free embryonated chicken eggs through the allantoic cavity [36,52]. After incubation (72 h at 37 °C), eggs were placed at 4 °C for about six hours. Harvested allantoic fluid was prepared by centrifugation at 12,000× *g* for 3 min. It was subsequently tested for the presence of hemagglutinating viruses with 0.5% chicken red blood cells (cRBCs) [53], which were prepared in sterile isotonic phosphate-buffered saline (PBS) at pH 7.2, as described previously [54]. Hemagglutination inhibition (HI) tests were also performed according to standard protocols [53]. 

### 2.5. Statistical Analysis

All statistical analysis was performed using the Prism V6.01 statistical software (GraphPad, San Diego, CA, USA).

## 3. Results

A total of 169 pooled samples were tested (Table 1). Of the 169 pooled samples, 16 (9.5%) were positive for the M gene (Table 1).

### 3.1. Identification of Avian Influenza Virus (AIV) Type A

To determine the prevalence of AIV type A, RT–qPCR assays were performed. Out of 844 samples, 21 were positive for AIV Type A, and the positive results were confirmed by both HA and HI assays (Table 3).

### 3.2. Prevalence of Avian Influenza Type A (AIV) in Semi-Scavenging Domestic Ducks

The overall prevalence of AIV Type A in apparently healthy semi-scavenging ducks from the seven wintering sites was 2.5% (*n* = 21). The site- and district-specific prevalence values of AIV H5, AIV H7, AIV H9, and other AIV H types are presented in Table 3. For Sunamgong district, the prevalence was 3.15%, followed by 3% for Moulavibazar, and 0.5% for Sylhet. For the sites, Tanguar haor had the highest prevalence of 4.7%, while Medholbill and Sathbilabill had no viral detection, as determined by different assays. Table 3 lists prevalence rates for other sites. The AIV prevalence rates also varied across seasons, with a prevalence rate of 4.1% in the winter, 2.5% in the summer, and 0.7% in the rainy season (Table 4). The most prevalent subtype was H9, which circulated in the winter (1.7%), the summer (1.1%), and the rainy season (0.4%). The H5 subtype circulated only in the winter (1.4%) and the summer (0.4%), but not in the rainy season. The H7 subtype was absent in all three seasons. Other subtypes circulated at different rates in the three seasons. The prevalence rates were 1% in the winter, 1.1% in the summer, and 0.4% in the rainy season (Table 4).

The M-gene-positive samples (*n* = 21) were subtyped for H5, H7, and H9 by RT–qPCR assay. Results revealed the predominance of H9 (9/21; 42.86%) over the H5 subtype (5/21; 23.8%). The H7 subtype was not detected. Other H subtypes represented 7/21 (33.3%) of the samples. The overall prevalences of H5, H7, and H9 were 0.6%, 0%, and 1.1%, respectively, while 0.8% of the samples were positive for other H genes (Table 4). More site-specific prevalence rates are shown in Table 3 and Table 4.

## 4. Discussion

Bangladesh is a low-lying riverine country where the confluence of the rivers and tributaries, haors, bills, lagoons, and marshland makes it suitable for duck rearing. Moreover, the Sylhet division has many of the popular haors and bills (lowland depression wetlands), including one of the largest water bodies in Bangladesh, the Hakaluki haor. These sites are important sanctuaries for migratory birds and are shared by millions of nomadic birds as well as domestic semi-scavenging ducks, especially during the winter. The country also lies along the Central Asian and East Asian migratory bird flyway [44]. Based on the previous information, Bangladesh can be at risk of infection, reinfection, and spread of AIV. 

In the current study, we demonstrated evidence of the cocirculation of influenza H5, H9, and other nonspecific influenza viruses in Bangladesh. The finding that viruses were recovered from 2.5% of apparently healthy semi-scavenging ducks in this study has negative public health implications. First, the apparently healthy but infected ducks could be easily missed during active surveillance because the focus will most likely be on apparently sick birds. The infected but apparently healthy ducks can, thus, spread the viruses among the highly susceptible flocks of chickens and other birds during intense contact. In addition, because the ducks are allowed to scavenge around, the spatiotemporal spread of pathogens is likely. In Bangladesh, the average number of ducks per farm varies from about 100 to more than 500. The spread of infection in one flock can compromise other flocks’ health due to the spatial distance that an infected scavenging flock can cover, thereby presenting broad spatiotemporal risks of transmission of the infection to other flocks. 

Moreover, many backyard farmers and small-scale farmers pay minimal attention to healthcare, biosecurity, and vaccinations. Ducks, chickens, and other livestock are typically housed together in the same location. Ducks are considered to be natural reservoirs for both LPAI viruses [55] and HPAI viruses [46]. Although duck populations and nomadic free-range chickens are high in number in the Sylhet division of Bangladesh, most AIV surveillance activities have been focused on commercial poultry farms. Thus, it is important to design specific surveillance protocols to target scavenging duck populations and free-range chickens in Bangladesh. 

District-specific and site-specific differences were observed in this study. Overall, the AIV prevalence rates in poultry in the Sunamgong and Moulavibazar districts were higher than in the Sylhet division. Certain sites (haors) in the Sylhet and Sunamgong districts had higher AIV prevalence rates. These findings should help guide public health policies in Bangladesh. Sarker et al. reported a high prevalence of AIV in Mymensingh and Sylhet divisions (60.73% and 47.73% seropositive samples, respectively) [44]. Other studies reported a prevalence rate of 22.1% for AIV in cloacal swab samples of domestic ducks in the winter months of December to February [36]. Kim et al. reported that the H9 subtype (63.2%) was more frequent than the H5 subtype (21.6%) in live bird markets (LBMs) in Bangladesh [27]. A previous study reported an overall prevalence rate of 23.0% in birds at LBMs in Bangladesh [56]. The lower prevalence rates in this study can be attributed to the consideration of multiseasonal data rather than focusing on high-risk periods or highly susceptible subjects. Ansari et al reported a prevalence of 0.9% for the M gene in cloacal and oropharyngeal swabs of ducks and chickens based on combined sampling [57]. Another study on waterfowl in Bangladesh reported a prevalence rate of 4.4% for AIV Type A, including a 1.9% positivity rate for the H5 subtype [28].

Migratory birds may have heightened risks of AIV infections in Bangladesh. During the overwintering period, migratory birds interact with domestic ducks at the wetlands. The isolation rates, and consequently, risks of infection increase for the H5, H9, and other AIV subtypes. The overall prevalence rates were highest in the winter (4.1%), less in the summer (2.5%), and much less in the rainy season (0.7%) (Table 4). Interestingly, this seasonal pattern is akin to the pattern in reports in which the prevalence of the HPAI H5N1 was higher in the winter season in Southeast Asian countries [57,58]. This may indicate an active role of migratory waterfowls in the transmission and spread of different subtypes of influenza viruses into domestic semi-scavenging ducks when sharing the wintering sites in Bangladesh. As a result of getting exposed to AIV from migratory birds, semi-scavenging ducks can subsequently transmit the viruses to the commercial chicken, backyard chicken, or even to humans without showing clinical signs of an AIV infection. It should be noted that a prevalence of 16.5% has been reported for H9 subtypes in the retail bird markets in Bangladesh [56]. In this study, the H9 viral subtype was more dominant. In a previous report, the H9 subtype was more common in migratory birds (56%) than in household ducks (38.3%) [59]. This could enhance the chances of AIV reassortment and the evolution of novel AIV strains. While the H9 subtype is the predominantly circulating subtype in the Sylhet division of Bangladesh, other AIV subtypes including but not limited to the H5 subtype exist. In winter, the H5 and H9 subtypes are more dominant than other subtypes in the wetlands of Chattogram and Sylhet, regardless of the species of the birds [59]. A less than 0.1% prevalence of the H5 subtype was reported in semi-scavenging duck populations in Bangladesh [36] and a similar percentage was also reported in the LBMs of Bangladesh [56]. The identification of high prevalence rates for AIV from wintering sites in Bangladesh calls for more intensive surveillance to reduce the threats of pathogenic AIV. This is especially relevant since the migratory bird flyway routes contribute to the wide dispersal of the influenza virus, and Bangladesh falls squarely along these routes [60]. Thus, it is important to study the specific contributions of these flyways to infections and outbreaks in Bangladesh and other countries along the same routes.

It is noteworthy that both HPAI and LPAI viruses are endemic in Bangladesh and have caused great economic losses. The cocirculation both in a reservoir host species, such as semi-scavenging ducks, poses risk to human health. This is because ducks may serve as reservoirs and reassortment vessels for the exchange of genetic materials between the influenza viruses which can cause the reemergence of new subtypes that have the potential of causing human infections or pandemics. In order to curtail the spread of different subtypes of AIV, there is a need for continuous monitoring via serological and virological surveillance and improved diagnostics for circulating viruses in Bangladesh and elsewhere in the world. 

## Figures and Tables

**Table 1:** The number of cloacal samples collected from different districts in Bangladesh.

District	Wintering Site	Sample Classifications				M-Gene-Positive Pooled Samples (*n*)	M-Gene-Positive Pooled Samples (%)
		Cloacal Samples			Pooled Samples (*n*)		
		Collected Samples (*n*)	Discarded Samples (Low Quality) (*n*)	Total Tested Samples (*n*)			
Sylhet	Tamabill	70	0	70	14	1	7.1
Sylhet	Sathbilabill	70	0	70	14	0	0
Sylhet	Medholbill	65	5	60	12	0	0
Sunamgong	Tanguar haor	150	0	150	30	5	16.7
Sunamgong	Khorchar haor	152	2	150	30	2	6.7
Sunamgong	Shonir haor	150	6	144	29	3	10.3
Moulavibazar	Hakaluki haor	203	3	200	40	5	12.5
All wintering sites (combined)		860	16	844	169	16	9.5

**Table 2:** Primer and probe oligonucleotides for RT–qPCR.

Type	Oligo Name	Sequence (5′-3′)	Final Conc. (nM)	Reference
**AIV type A**				[48]
Forward	IVA D161M	AGATGAGYCTTCTAACCGAGGTCG	900	
Reverse 1	IVA D162M1	TGCAAAAACATCYTCAAGTCTCTG	225	
Reverse 2	IVA D162M2	TGCAAACACATCYTCAAGTCTCTG	225	
Reverse 3	IVA D162M3	TGCAAAGACATCYTCAAGTCTCTG	225	
Reverse 4	IVA D162M4	TGCAAATACATCYTCAAGTCTCTG	225	
Probe	IVA Ma	FAM-TCAGGCCCCCTCAAAGCCGA-TAMRA	250	
**H5 duplex**				[48]
Forward 1	IVA D148H5	AAACAGAGAGGAAATAAGTGGAGTAAAATT	675	
Forward 2	IVA D204	ATGGCTCCTCGGRAACCC	675	
Reverse 1	IVA D149H5	AAAGATAGACCAGCTACCATGATTGC	675	
Reverse 2	IVA D205	TTYTCCACTATGTAAGACCATTCCG	675	
Probe 1	IVA H5A	FAM-TCAACAGTGGCGAGTTCCCTAGCA-TAMRA	300	
Probe 2	IVA D215	FAM-ATGTGTGACGAATTCMT-MGB-NFQ	300	
**H7**				[49]
Forward	IAV-HA7-1593	AYA GAA TAC AGA TWG ACC CAG T	20,000	
Reverse	IAV-HA7-1740	TAG TGC ACY GCA TGT TTC CA	20,000	
Probe	AIV-HA7-1649	FAM-TGG TTT AGC TTC GGG GCA TCA TG-BHQ1	2500	
**H9**				[50]
Forward	H9 Fwd	ATGGGGTTTGCTGCC	900	
Reverse	H9 Rev	TTATATACAAATGTTGCAC(T)CTG	900	
Probe	H9 Probe	TTCTGGGCCATGTCCAATGG	250	

**Table 3:** Prevalence of AIV Type A from cloacal swabs.

Study Area (District)	Wintering Sites	Total Number of Samples	RT–PCR-Positive Samples of AIV Type A	HA/HI-Positive Samples	Prevalence of AIV Type A (%) in Each Site	Prevalence of AIV Type A (%) in Each District
Sylhet	Tamabill	70	1	1	1.4	0.5 (Sylhet)
Sylhet	Sathbilabill	70	0	0	0	
Sylhet	Medholbill	60	0	0	0	
Sunamgong	Tanguar haor	150	7	7	4.7	3.15 (Sunamgong)
Sunamgong	Khorchar haor	150	3	3	2.0	
Sunamgong	Shonir haor	144	4	4	2.78	
Moulavibazar	Hakaluki haor	200	6	6	3.0	3.0 (Moulavibazar)
**Total**		**844**	**21**	**21**	**2.5**	**2.5**
**Study Area** **(District)**	**Wintering sites**	**AIV Type A positive**	**AIV H5 positive (%)**	**AIV H7 positive (%)**	**AIV H9 positive (%)**	**Other AIV H positive (%)**
Sylhet	Tamabill	1	0 (0)	0 (0)	1 (100)	0 (0)
Sylhet	Sathbilabill	0	0 (0)	0 (0)	0 (0)	0 (0)
Sylhet	Medholbill	0	0 (0)	0 (0)	0 (0)	0 (0)
Sunamgong	Tanguar haor	7	2 (28.6)	0 (0)	2 (28.6)	3 (42.8)
Sunamgong	Khorchar haor	3	1 (33.3)	0 (0)	1 (33.3)	1 (33.3)
Sunamgong	Shonir haor	4	1 (25.0)	0 (0)	2 (50.0)	1 (25.0)
Moulavibazar	Hakaluki haor	6	1 (16.7)	0 (0)	3 (50.0)	2 (33.3)
Rel. prevalence in the positive samples		21	5 (23.8)	0 (0)	9 (42.9)	7 (33.3)
**Overall prevalence (*n* = 844)**			**5 (0.6)**	**0 (0)**	**9 (1.1)**	**7 (0.8)**

**Table 4:** Seasonal Prevalence of AIV Type A and different subtypes in semi-scavenging ducks.

Season	District	Number of Samples	Number of AIV Isolates									
			AIV Type A Positive	Seasonal Prevalence (%)	AIV H5 Positive	Seasonal Prevalence (%)	AIV H7 Positive	Seasonal Prevalence (%)	AIV H9 Positive	Seasonal Prevalence (%)	Other Subtype Positive	Seasonal Prevalence (%)
Winter (Nov-Feb)	Sylhet	70	1	4.1 (W)	0	1.4 (W)	0	0 (W)	1	1.7 (W)	0	1.0 (W)
	Sunamgong	150	8		3		0		3		2	
	Moulavibazar	70	3		1		0		1		1	
Summer (Mar-Jun)	Sylhet	65	0	2.5 (S)	0	0.4 (S)	0	0 (S)	0	1.1 (S)	0	1.1 (S)
	Sunamgong	150	5		1		0		2		2	
	Moulavibazar	65	2		0		0		1		1	
Rainy (Jul-Oct)	Sylhet	65	0	0.7 (R)	0	0 (R)	0	0 (R)	0	0.4 (R)	0	0.4 (R)
	Sunamgong	144	1		0		0		0		1	
	Moulavibazar	65	1		0		0		1		0	
**Total**		**844**	**21**	**2.5**	**5**	**0.6**	**0**	**0**	**9**	**1.1**	**7**	**0.8**

## Data Availability

All relevant data are included within the manuscript.
